# The Risk of Depression in Patients With Cholelithiasis Before and After Cholecystectomy: A Population-based Cohort Study

**DOI:** 10.1097/MD.0000000000000631

**Published:** 2015-03-13

**Authors:** Te-Chun Shen, Hsueh-Chou Lai, Yu-Jhen Huang, Cheng-Li Lin, Fung-Chang Sung, Chia-Hung Kao

**Affiliations:** From the Graduate Institute of Clinical Medicine Science, College of Medicine, China Medical University (T-CS, H-CL, F-CS, C-HK); Division of Pulmonary and Critical Care Medicine (T-CS); Division of Gastroenterology and Hepatology, Department of Internal Medicine (H-CL); Department of Psychiatry (Y-JH); Management Office for Health Data (C-LL); and Department of Nuclear Medicine and PET Center, China Medical University Hospital, Taichung, Taiwan (C-HK).

## Abstract

The association between cholelithiasis and depression remains unclear. We examined the risk of depression in patients with cholelithiasis.

From the National Health Insurance population claims data of Taiwan, we identified 14071 newly diagnosed cholelithiasis patients (4969 symptomatic and 9102 asymptomatic) from 2000 to 2010. For each cholelithiasis patient, 4 persons without cholelithiasis were randomly selected in the control cohort from the general population frequency matched by age, sex, and diagnosis year. Both cohorts were followed up until the end of 2011 to monitor the occurrence of depression. Adjusted hazard ratios (aHRs) of depression were estimated using the Cox proportional hazards model after controlling for age, sex and comorbidities.

The overall incidence rates of depression were 1.87- and 1.83-fold greater in the symptomatic and asymptomatic cholelithiasis subcohorts than in the control cohort (incidence, 10.1 and 9.96 vs 5.43 per 1000 person-years, respectively). The multivariable Cox proportional hazards regression analysis revealed higher variable-specific aHRs in women than in men, in younger patients than in older patients, and in those without comorbidities than in those with any comorbidity. Cholecystectomy reduced the hazard of developing depression with aHRs of 0.79 (95% confidence interval [CI] 0.62–0.99) for symptomatic cholelithiasis patients and 0.76 (95% CI 0.60–0.96) for asymptomatic patients.

Patients with cholelithiasis are at a higher risk of developing depression than the general population. Patients could be benefited from cholecystectomy and have the hazard of developing depression significantly reduced.

## INTRODUCTION

Involving the presence of stones in the gallbladder, cholelithiasis is generally a chronic condition with the prevalence varied markedly among ethnic populations, 4.6% in a recent Chinese study, or 5% to 10% in Asian populations.^1,2^ There are 2 major types of gallstones: cholesterol stones and pigment stones. Unlike in Western populations, Asian populations are prevalent with pigment stones.^3^ Patients with gallstones can be symptomless or suffered from various clinical complications, such as biliary colic, obstructive jaundice, pancreatitis, cholecystitis/cholangitis, and even gallbladder cancer. Studies have found many chronic diseases may be comorbid depression.^4–5^ A study using the World Health Survey data found that the prevalence of comorbid depression increased with the number of chronic disease increased, from 9.3% to 23.0%, in 245,404 participants from 60 countries.^6^

Cholelithiasis is an asymptomatic chronic medical condition for most of patients. Some complications may occur to gallstone patients and become the source of stress for them, including those with biliary colic, which is a type of recurrent pain as the stone transiently obstructs the cystic duct and the gallbladder contracts.^7^ Several studies have investigated the relationship between depression and biliary diseases. An early German study showed that women with biliary calculi (51 patients) were at higher risks for not only depression but also emotional instability than controls (74 participants).^8^ However, other studies failed to reach a statistically significant association or yielded inconclusive results.^9–10^ Most of these studies have the limitation of small sample sizes. To the best of our knowledge, no study has ever used a large population data to investigate the relationship. The present study attempts to determine the risk of depression in patients with cholelithiasis using a nationwide population-based data obtained from the Taiwan National Health Insurance, which has been used in a number of studies.^11–13^

## MATERIALS AND METHODS

### Data Source

Established in 1996, the insurance system has provided health care coverage to more than 99% of the 23.72 million populations in Taiwan since 1998. The National Health Research Institutes (NHRI) is responsible to maintain the insurance data and established data files for research. Patient identifications are scrambled before releasing the data to users in order to protect the privacy of insured people. We obtained a representative subset of Longitudinal Health Insurance Database 2000 (LHID 2000), consisting of claims data from 1996 to 2011 for one million randomly selected people. The claims data included information on demographic data, dates of clinical visits, diagnostic codes, and details of prescriptions, and treatment procedures including surgeries. Diseases are coded using the *International Classification of Diseases* (ICD) -9-CM, 2001 edition. This study was exempted from full ethical review by the China Medical University and Hospital Research Ethics Committee (IRB permit number: CMU-REC-101–012).

### Study Cohorts

We selected new patients aged 20 years and older diagnosed with symptomatic cholelithiasis (ICD-9-CM code 574.0, 574.1, 574.3, 574.4, 574.6, 574.7, 574.8) and asymptomatic cholelithiasis (ICD-9-CM code 574.2, 574.5, 574.9), from 2000 to 2010 as the cholelithiasis cohort.^14^ The diagnosis date was designated the index date. Control subjects were randomly selected from the pool of same population without cholelithiasis. Four controls were selected frequency-matched by age (every 5-year span), sex, and year of cholelithiasis diagnosis. Individuals younger than 20 years, with a history of depression disorders (ICD-9-CM code 296.2, 296.3, 300.4, and 311) at the baseline and/or without information on birth date and sex, were excluded from both cohorts.

### Cormobidity and Outcomes

Both the cholelithiasis and control cohorts were followed until a diagnosis of depression was made or they were censored for loss to follow-up, withdrawal from the insurance program, or the date of December 31, 2011 was reached, whichever occurred first. The comorbidities investigated in this study were hypertension (ICD-9-CM codes 401–405), diabetes mellitus (ICD-9-CM code 250), hyperlipidemia (ICD-9-CM code 272), stroke (ICD-9-CM codes 430–438), asthma (ICD-9-CM code 493), chronic liver disease and cirrhosis (CLD) (ICD-9-CM code 571), and kidney disease (KD) (ICD-9-CM codes 580–589).

### Statistical Analysis

Chi-square test and *t* test were used to examine differences between cholelithiasis and control cohorts for categorical and continuous variables, respectively. The cumulative incidence of depression between the 2 cohorts was plotted by Kaplan–Meier method and the difference was tested by log-rank test. The incidence rates of depression were also calculated for the cholelithiasis cohort, for its subgroups (symptomatic and asymptomatic subcohorts), and for the control cohort by sex, age, and comorbidity. Cox proportional hazards regression analysis was used to assess the hazard ratio (HR) and 95% confidence interval (CI) of depression associated with cholelithiasis, compared with controls. Multivariable model was used to estimate the adjusted HR (aHR), controlling for covariates that were significant in the univariable model, including age, sex, and comorbidities of hypertension, diabetes, hyperlipidemia, stroke, asthma, CLD, and KD. Data analysis also evaluated the hazard of developing depression for cholelithiasis patients receiving cholecystectomy. Data analysis further measured mean years from the baseline to depression diagnosed for both cohorts and for symptomatic and asymptomatic patients associated with cholecystectomy. All statistical analyses were performed using SAS 9.3 software (SAS Institute, Cary, NC, USA) for Windows. The level of significance was set at 0.05 using a 2-tailed test.

## RESULTS

**FIGURE 1:**
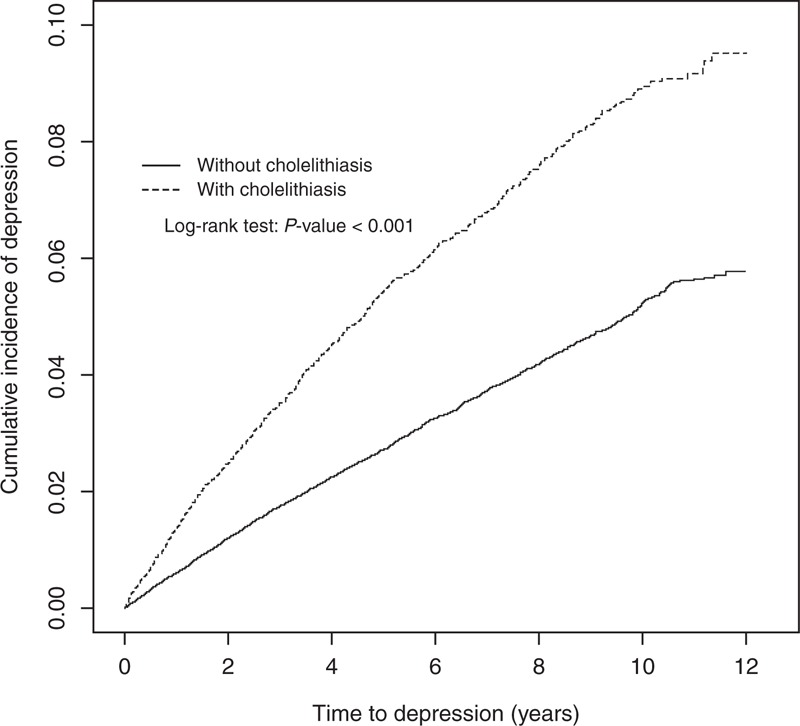
Cummulative incidence of depression in cholelithiasis cohort and control cohort.

We established a cholelithiasis cohort with 4969 symptomatic cases and 9102 asymptomatic cases, and a control cohort of 56,284 subjects without cholelithiasis (Table 1). There were more women and younger individuals, with mean ages of 56.0 (SD 15.7) years in the cholelithiasis cohort and 55.5 (SD 16.0) years in the controls. Comorbidities were more prevalent in the cholelithiasis cohort. The mean follow-up periods were shorter in the asymptomatic cholelithiasis subcohort (6.00 ± 3.29 years) than in the symptomatic cholelithiasis subcohort (6.17 ± 3.31 years) and the control cohort (6.22 ± 3.28 years). The Kaplan–Meier plot shows the cumulative incidence of depression in cholelithiasis patients was approximately 3.7% higher than that in controls (log-rank test *P* < 0.0001, Figure 1) after a 12-year follow-up.

**TABLE 1:**
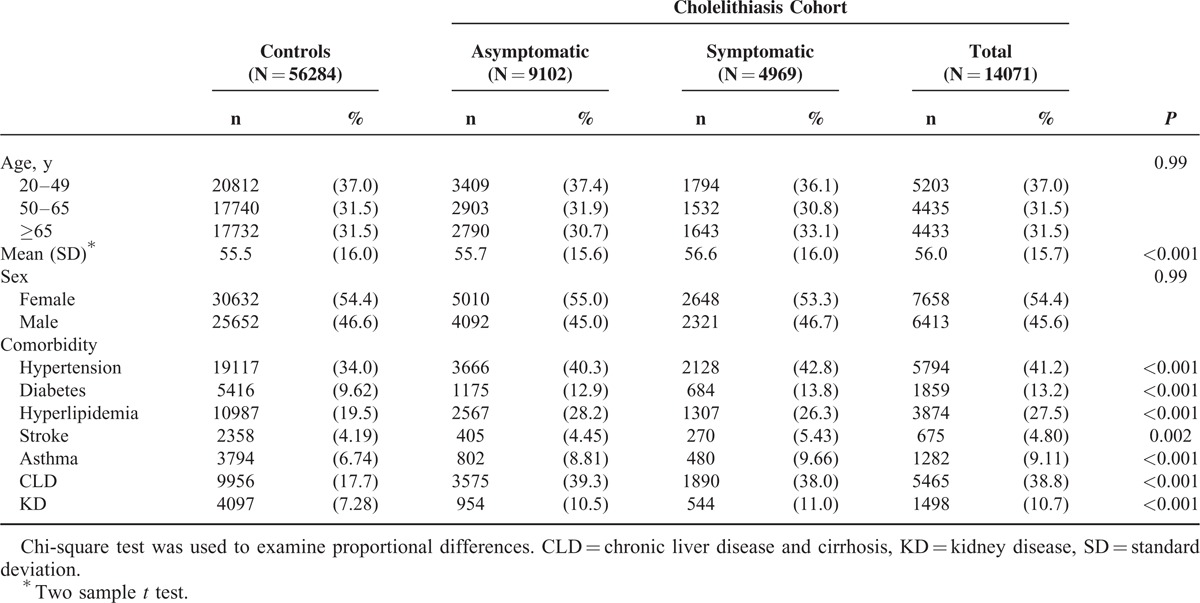
Comparison of Demographics Status and Comorbidity Between Cholelithiasis Patients and Controls

The incidence rates were 10.1, 9.96, and 5.43 per 1000 person-years in the subcohorts of symptomatic cholelithiasis and asymptomatic cholelithiasis, and the control cohort, respectively (Table 2). The multivariable Cox method estimated aHRs of developing depression were 1.71 (95% CI 1.51–1.93) for the symptomatic cholelithiasis subcohort and 1.68 (95% CI 1.66–2.01) for asymptomatic cholelithiasis subcohort, compared with the control cohort. The depression incidence was greater in women than in men in the 3 cohorts. The depression incidence increased with age in the 3 cohorts, but the age-specific hazard ratio was the greatest for the youngest group (HR = 2.00, 95% CI 1.62–2.46 for symptomatic cholelithiasis subcohort and HR = 1.85, 95% CI 1.56–2.19 for asymptomatic cholelithiasis subcohort). The incidence of depression was also higher in subjects with comorbidity than those without comorbidity.

**TABLE 2:**
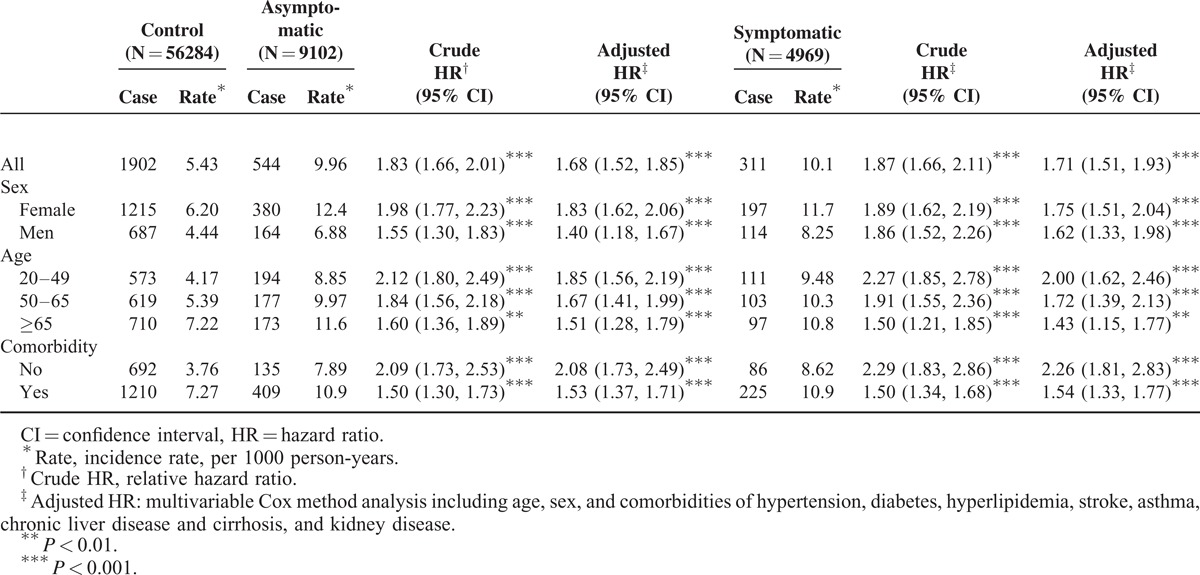
Cox Method Measured Incidence Densities and HR of Depression by Sex, Age, Comorbidity and Study Cohort

Table 3 shows that cholecystectomy reduced the incidence of depression for 21.5% in symptomatic cholelithiasis patients and for 23.9% for asymptomatic patients. The Cox method estimated aHRs were 0.79 (95% CI 0.62–0.99) and 0.76 (95% CI 0.60–0.96), respectively, compared with those without cholecystectomy. Table 4 shows that the occurrence of depression was approximately 0.4 years shorter (*P* < 0.001) in cholelithiasis patients than in controls. Cholecystectomy delayed the occurrence of depression for near 1.5 years in symptomatic cholelithiasis patients, compared with those without the surgery. The corresponding effect of surgery was near 1.2 years for asymptomatic cholelithiasis patients.

**TABLE 3:**
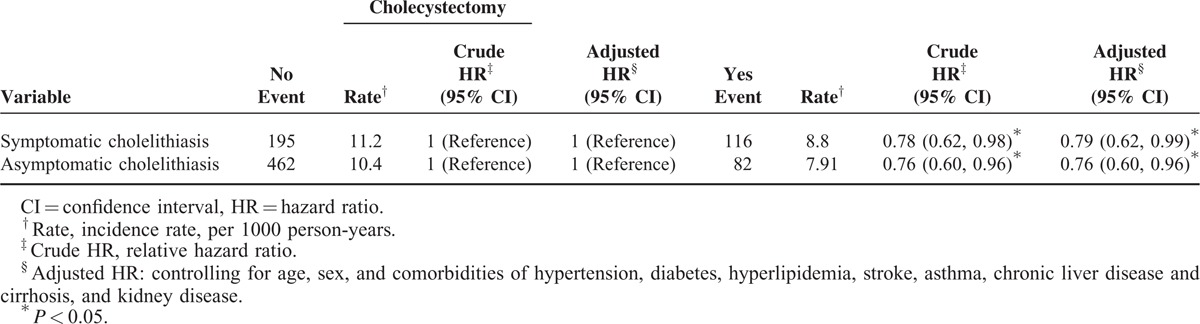
Incidence Rates and HRs of Depression in Cholelithiasis Patients With and Without Cholecystectomy

**TABLE 4:**
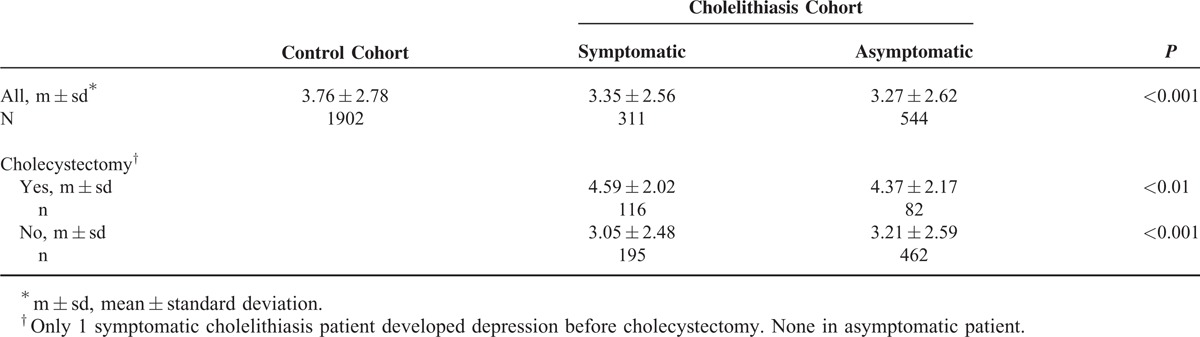
Mean Years From Baseline to Depression Development in Association With Cholecystectomy Among Those Who Had Developed Depression

## DISCUSSION

The relationship between cholelithiasis and subsequent depression risk has not been evaluated using population-based data in previous studies. Using the nationwide insurance claim data, we have identified a near 80% higher incident depression in patients with cholelithiasis than in the general population without the disease. We also found that females and individuals of older age had a higher incidence of depression in both cohorts. However, variable-specific aHRs showed that the depression hazards were greater in women than in men, in younger than in older patients, and in those without comorbidities than in those with comorbidity. Among population without comorbidities, the hazard of depression was >2-fold higher in cholelithiasis patients than in controls. Comorbidities increased the risk further. The findings indicate that cholelithiasis contributes a greater risk to patients in the development of depression than comorbidities do. We also found that cholecystectomy could reduce the depression risk significantly for >20%, reflecting that the surgery may reduce the threat of gallstones.

In the present study, we attempted to evaluate the difference of depression risk between symptomatic and asymptomatic groups among cholelithiasis patients. The incidence of depression was only 1.4% higher ([10.1–9.96]/9.96 per 1000 person-years) in the symptomatic group than in the asymptomatic group. Similar patterns appeared in the detailed sex, age, and comorbidity-specific incident differences between symptomatic and asymptomatic groups. However, the age-specific incidence was reversely slightly higher in asymptomatic group than in symptomatic group for the older ages. These findings suggest that the risk of depression development is not much less for asymptomatic patients than for symptomatic patients. There are other factors involved in the development of depression in addition to the presence of symptoms.

Depression has become an important worldwide health problem because of the high lifetime prevalence. Ustün et al^15^ reported it may account 12% of all total years lived with disability. The comorbidity of depression in patients with chronic diseases is a well-recognized concern. Studies have shown that there is an increased risk of developing depression in people with ≥1 chronic diseases.^16–17^ With the fast growth in the elderly population and associated increase in the prevalence of chronic illnesses, a concomitant rise in the prevalence of depression is expected.^6^ This scenario is well illustrated in our study.

Other mechanisms explaining the correlation between cholelithiasis and depression have been described. Inflammation may be one of the key factors. Previous study showed that immune activation and treatment with cytokines may induce depression symptom.^18–19^ Studies show the levels of cytokines are higher in the circulation in depressive patients.^20–23^ The peripheral levels of interleukin 6 (IL-6), tumor necrosis factor alpha, and IL-1b are increased. These increased cytokines levels can be reversed by antidepressant treatment, which evidently shows the link.^22,23^ Moreover, pain is another essential factor in the development of depression.^24^ A pooled analysis of multiple studies showed the prevalence of pain is as high as 65% on average among depressed patients.^25^

An earlier epidemiologic study on pain complaints confirmed an increased odd for developing depressive symptoms in patients with chronic pain.^26^ Patients with more than one pain complaints are 3 to 5 times more likely to develop depression than those without pain. Neuroimaging studies have reported that chronic pain can alter the brain structure and function.^27^ Patients with symptomatic cholelithiasis may suffer from pain and threat of complications. However, the definitive correlative mechanisms between cholelithiasis and depression remain largely unknown, particularly for those with asymptomatic cholelithiasis.

The option of cholecystectomy remains controversial, particularly for patients with asymptomatic gallstones. The World Gastroenterology Organization has not yet recommended cholecystectomy as benefit to patients with asymptomatic gallstones or to patients with a single attack of uncomplicated gallstone pain.^28^ The risk of the operation outweighs the complications if stones remain untouched. In brief, the main concerns include it is difficult to determine cholecystectomy may resolve the symptoms particularly the biliary symptom; 1% to 2% of post-cholecystectomy patients suffer from chronic diarrhea, requiring bile acid sequestrants for management; the risk-benefit evaluation may not prove cholecystectomy as cost-effective; the prophylactic surgery is expensive. However, cholecystectomy could be indicated for patients living in remote areas and high-risk areas, and for patients with immunosuppressive condition and with calcified porcelain gallbladder. In the present study, our data showed cholecystectomy could significantly reduce the risk of developing depression, even for patients with asymptomatic gallstone disease. Our data showed the average times from the baseline when gallstone was diagnosed to the occurrence of depression were both near 3.3 years in symptomatic and asymptomatic subcohorts. Therefore, cholecystectomy could be a treatment recommendation even for asymptomatic patients at high risk.

A major strength of our study is the use of population-based data with results highly representative of the general population. However, certain limitations should be considered. First, this study used the ICD-9-CM algorithm to define cholelithiasis, depression, and comorbidities. The diagnosis depends on the performance of clinical physicians. An ad hoc committee established by the insurance authority was in charge of monitoring the claims data to prevent errors and violations. In the present study, only disease with the diagnosis that had been reported in the claims data for at least twice within a year in the same ICD code was counted to improve the validity and accuracy of the diagnosis. Second, information on smoking habits, diet preference, occupation, and family history of systemic diseases was not available in the data analysis for confounding adjustments in the association determination between cholelithiasis and depression. Third, relevant clinical data such as depression rating scale, body mass index, serum laboratory data, imaging results, and pathology findings of the subjects were also unavailable in our study.

## CONCLUSION

Patients with cholelithiasis are at a higher risk of developing depression than the general population regardless of age, sex, and presence of comorbidities. Patients who underwent cholecystectomy could have a reduced risk of depression. As asymptomatic cholelithiasis is much more common than symptomatic cholelithiasis in the population, the asymptomatic cholelithiasis is no less important than the symptomatic cholelithiasis in the risk of developing depression.
